# Towards Functional Parts by Binder Jetting Calcium-Sulphate with Thermal Treatment Post-Processing

**DOI:** 10.3390/ma13173818

**Published:** 2020-08-29

**Authors:** María Ángeles Castro-Sastre, Ana Isabel Fernández-Abia, Janik Piep, Pablo Rodríguez-González, Joaquín Barreiro

**Affiliations:** 1 Department of Mechanical, Informatics and Aerospace Engineering, University of León, Campus de Vegazana, 24071 León, Spain; macass@unileon.es (M.Á.C.-S.); prodrg@unileon.es (P.R.-G.); jbarg@unileon.es (J.B.); 2 Bremen University of Applied Sciences, 28199 Bremen, Germany; jpiep@stud.hs-bremen.de

**Keywords:** additive manufacturing, binder jetting, calcium-sulphate, thermal treatment, post-processing

## Abstract

The objective of our research is to improve the properties of calcium-sulphate hemihydrate parts printed by binder jetting. In this paper, we show the thermal treatment results when using temperature time ramps on binder-jetted ceramic parts without infiltrating. The results show that the mechanical properties of printed parts are improved substantially. Two different thermal cycles were investigated for their effect on the dehydration process of CaSO_4·_½H_2_O using infrared analysis. The thermal-treated samples were compared with respect to porosity, surface roughness, compression strength and dimensional and weight variation. The proposed thermal treatment significantly improves the compression strength in a short time, guaranteeing dimensional stability while providing a good surface. This improvement in mechanical properties offers a great chance for using binder-jetted parts as functional components, for example, in the casting field or the medical sector (scaffolds).

## 1. Introduction

The additive manufacturing (AM) has had a major influence on the manufacturing of many technical products for several years now. The wide variety of currently available processes makes it possible to print a diverse variety of materials [1] and fully functional parts [2]. One of the categories of processes defined in the standards [3,4] is binder jetting. After over 25 years, this technology has been used to manufacture parts of various materials for different commercial applications [5,6]. In this group of processes, a micrometre-sized powder bed is available. A carrier with a microinjector printhead projects a binder mixture, with or without colour, that acts as a binding element for joining the powder by means of chemical reaction with it. The complete binder jetting process includes printing, setting, de-powdering and post-processing. However, these stages can vary depending on the raw material (metal, ceramic, polymer, etc.).

The properties of binder jetting printed parts depend on several factors: (i) operational parameters (printing speed, layer printing delay, layer thickness), (ii) powder properties (particle size, composition, humidity, flowability, wettability) and (iii) binder (concentration, activator content, binder type) [7,8]. In addition, it is also advisable to improve the properties of parts by applying post-processing, such as sintering, infiltration, thermal treatment or finishing [9]. Ziaee et al. [10] noted that post-processing is a very suitable technique to improve the properties of binder-jetted parts.

Researchers have worked hard in recent years in the aforementioned areas of interest to improve the technology and to achieve parts with better properties. Some researchers have focused on optimising operational parameters and strategies. Yao et al. [11] and Tsung-Jung et al. [8] studied different process parameters such us layer thickness, binder saturation and position of parts on the printer table for plaster powder. Suwanprateeb et al. [12] investigated the effect of layer thickness and binder saturation for the same material. Chen et al. [7], using the Taguchi method, studied the relationship between four important process parameters (layer thickness, printing saturation, heater power ratio and drying time) and the dimensions and surface roughness of binder-jetted parts.

Other authors studied the influence of binder jetting parameters on the mechanical properties of the parts. Shrestha et al. [13] used the Taguchi method in order to improve transverse rupture strength (TRS). The printing parameters analysed were binder saturation, layer thickness, roll speed and feed-to-powder ratio. Asadi et al. [14] and Farzadi et al. [6] also used the design of experiments approach in order to determine the optimal printing parameters for making calcium-sulphate-based scaffold prototypes. The printing parameters evaluated in their studies were layer thickness, delay time and specimens’ build orientation. Coniglio et al. [15] also studied the influence of these parameters (job box positions, print resolution and re-coater speed) using design of experiments to analyse their effect on mould strength, permeability and minimising anisotropic behaviour. Castliho et al. [16] studied the topological optimisation and the influence of printing direction on the mechanical properties, permeability and stiffness of scaffolds made of tricalcium phosphate powder by binder jetting. The results determined that the low-temperature three-dimensional (3D) printing process is a viable option to manufacture synthetic scaffolds. Miyanaji et al. [17] showed that printing speed influences the dimensional precision and the saturation level of binder-jetted parts.

Other authors focused on studying features of the powder and binder raw materials. In Reference [18], the authors experimentally investigated the influence of powder characteristics on the quality of the binder jetting manufacturing parts. They studied the effect of particle sizes and size distributions on surface roughness and densities for green and sintered parts.

Finally, other authors have studied the post-processing. Commonly, hardware, software and materials in industrial machines are difficult to change and modify. Manufacturers preserve these elements to provide repeatability and accuracy in their machines. Therefore, most research cannot significantly vary the parameters or the printing operation and, therefore, the results are very constrained. In this context, post-processing acquires special importance to improve the printed part properties. Without post-processing the binder jetting printed parts, there is not a product. It is a so-called “green-body” that requires drying, sintering or infiltration.

Some authors have focused on infiltration post-processing. Ocaña et al. [19] studied the feasibility of making plaster moulds using binder jetting technology to apply in casting processes. The moulds were post-treated using different infiltrates and post-processing parameters. The aim was to achieve a process cost lower than metallic AM processes. Castro et al. [20] analysed the influence of different infiltration materials and related research using gaseous and liquid phases. Watters et al. [21] developed a post-curing process for the parts in order to increase the depth of infiltration and achieve more resistant parts. The material was calcium-sulphate hemihydrate. The same research was done in References [22,23] but for orthodontic models. Maravola et al. [24] used an epoxy polymer for infiltrating tooling moulds based on silica sand and zirconia produced via binder jetting. Ayres et al. [25] investigated various infiltrates and different infiltration methods to improve mechanical strength and temperature performance of plaster binder jetting parts. Infiltrates based on epoxy resins and cyanoacrylate were compared, including the use of hot and wet vacuum.

Miyanaji et al. [26] developed a model to estimate the optimal saturation levels for green printed parts. The model is based on the interactions between binder and powder in the equilibrium state. James et al. [27] studied the nanoparticle infiltration during the binder jetting process using molecular dynamics simulation techniques. They carried out infiltration simulations at different temperatures, concluding that the infiltration kinetics is strongly affected by the infiltration temperature.

Another post-processing method, complementary to infiltration, is thermal treatment in a furnace. Several works have revealed that curing parameters (temperature and time) modify the properties of printed parts, such as strength and permeability. However, not many research results are currently available for the thermal treatment of three-dimensional (3D)-printed calcium sulphate, especially considering its influence on mechanical properties.

Mitra et al. [28] studied the relationship between the amount of binder present in a binder jetting mould and the bending strength and the permeability. The modification of these properties is related to reactions that the binder undergoes under certain conditions of temperature and time. The increase in strength may be associated to evaporating the solvent, which origins shortening and hardening of resin bridges. Bassoli and Atzeni [29] carried out an experimental study to evaluate the mechanical and dimensional changes induced by different thermal treatments. They concluded that curing time is almost irrelevant for compression strength, whereas the temperature has a significant effect. They also concluded that dimensional accuracy is almost independent of the thermal treatment applied. Ledingham et al. [23] studied the improvement in mechanical properties and dimensional precision of calcium-sulphate dental models after applying different post-treatments. They applied treatments at high heat (250 °C for 30 min), low heat (150 °C for 30 min) and Epsom salt treatment. Ocaña et al. [19] applied thermal treatments to calcium-sulphate hemihydrate moulds using temperature time ramps. Three cycles were studied as a function of the infiltrate used (ethyl silicate, Levasil, Ludox SK, Aerodisp, Ticoat-N and zirconium acetate). In this research, the material was not in the raw state (infiltrates were used) and temperatures were very high as a function of the infiltrate. In this temperature range, the base material (calcium-sulphate hemihydrate) degrades. Zhou et al. [30] and Asadi-Eydivand et al. [31] tested 3D-printed scaffolds made of calcium-sulphate for use as bone tissue. These scaffolds were heat-treated.

Despite these studies, none of these authors have tested using temperature time ramps for considering the phase changes in the powder and their effect on the binder and volatiles and how it influences the mechanical properties.

As already commented, one of the binder jetting processes most used is that which uses calcium-sulphate hemihydrate powder (CaSO_4_·½H_2_O) as the raw material. This process applies a binder solution that consists essentially of water with additives (furan wax, 2–pyrrolidone, etc.). This binder allows quicker plaster setting and hardening. However, this process is usually restricted to manufacturing aesthetic parts, ergonomic applications or models. Due to the fragility of parts obtained with this technique and material, it is not usually used for functional parts. Consequently, the applicability of these parts to engineering tasks is limited, as their mechanical properties are very poor.

The usual post-processing in these parts is to infiltrate to improve the mechanical strength. Manufacturers often recommend using soluble infiltrates, which penetrate the parts due to their high porosity. The manufacturers provide recommendations about using certain water-soluble sulphate, such as magnesium sulphate (MgSO_4_·7H_2_O), which make it possible to accelerate the setting of these hemihydrates. Other types of infiltrates include a solution of cyanoacrylate in water (c2–methoxy–2–ethyl–cyanoacrylate) or paraffin wax and epoxy resin (EX-401). However, as previously mentioned, several authors have investigated using alternative infiltrates to improve the properties of the parts. However, most of these studies only considered the infiltration process without applying any thermal treatment. Moreover, the few research studies that apply some thermal treatment carry it out on the printed material once it has been infiltrated.

Previous research has shown that the properties of printed plaster ceramic parts can have great potential but there is still a wide range for improving. Calcium-sulphate is one of the most commonly used materials in the binder jetting process. This material is commonly used for medical and dental applications. Also, calcium-sulphate can be used for foundry application to print moulds for non-ferrous castings. For this application, to post-process the mould is necessary to reduce the water content and to improve the strength, porosity and surface roughness. The aim of our work is to improve the properties of binder jetting printed parts by using thermal treatments before infiltration. As previously mentioned, only a few authors have tested this option [32]. Nevertheless, these authors do not test using temperature time ramps for considering the phase changes in the raw powder, its effect on the binder and volatiles and the influence in the mechanical properties. In Ocaña et al. [19], the temperature time ramps were conditioned by the infiltrate used; therefore, the scope of this research is very different from ours since in our case, we do not apply any infiltrate before the thermal treatment. In the next sections, the experimental procedure and the results are explained in detail.

## 2. Materials and Methods

### 2.1. Manufacturing Test Specimens and Raw Materials

**Figure 1:**
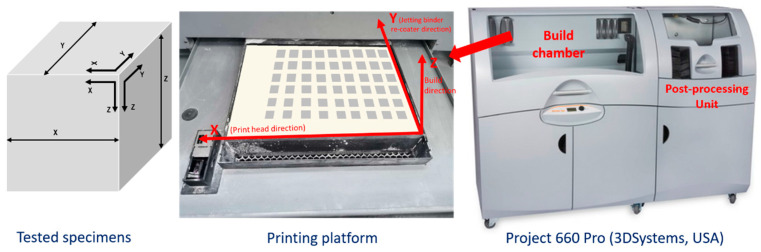
Printing machine, work platform and specimens printed.

The tested specimens were manufactured by binder jetting technology using a Project 660Pro machine (3DSystems, Rock Hill, SC, USA). This machine has a 600 × 540 dpi resolution, a 254 × 381 × 203 mm build volume, 0.1 mm layer thickness and 28 mm/h maximum vertical build speed. The printed specimens were 40 × 40 × 40 mm cubes. They were randomly placed throughout the work platform and were oriented according to the machine axis. Axis X is the print-head direction, axis Y is the re-coater direction and axis Z is the build direction, perpendicular to the printed layers. Figure 1 shows the machine used, the build chamber and the specimens printed, indicating the coordinate system.

The raw material used was calcium-sulphate hemihydrate (CaSO_4_·½H_2_O) with a solution of 2–pyrrolidone binder with a high water content. The composition of the two raw materials was studied in previous work using different analytical techniques [33]. The results using Raman spectrometry and X-ray diffraction show that the powder has all the species of the system CaSO_4_·H_2_O: gypsum, hemihydrate and anhydrite, with hemihydrate in its beta phase as the majority species. The results obtained from Inductively Coupled Plasma Optical Emisión Spectrometry (ICP-OES) show the same impurities in both raw materials. These impurities are aluminium, magnesium, potassium, sodium and silicon, and this is only in the powder material. Table 1 shows the chemical composition of impurities in the powder and binder.

### 2.2. Specimens Preparation

**Figure 2:**
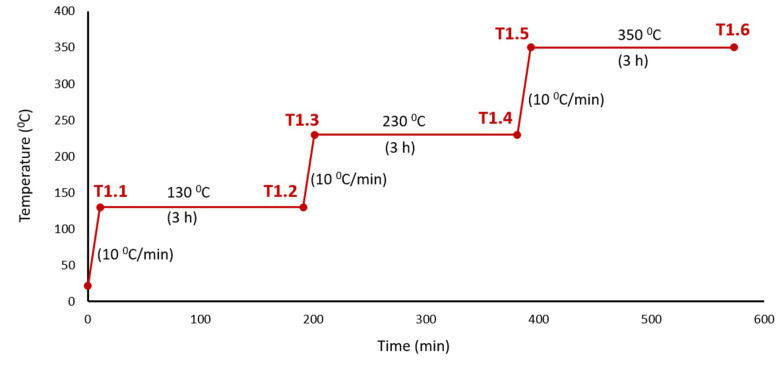
Thermal cycle of Experiment 1. The tested temperatures were 130, 230 and 350 °C with a 3 h holding time and 10 °C/min temperature increase.

**Figure 3:**
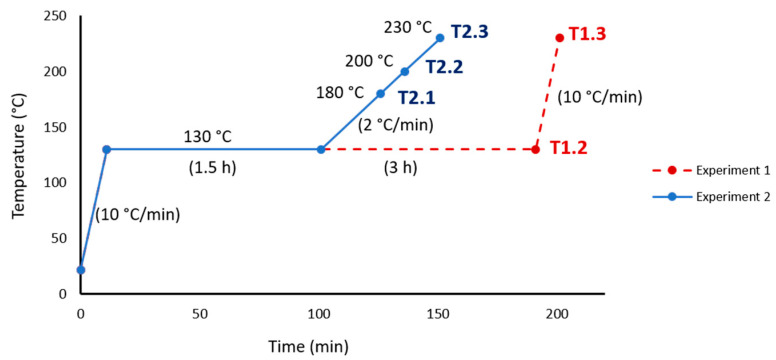
Thermal cycle of Experiment 2. Tested temperatures were 180, 200 and 230 °C without holding times. The first temperature increase was 10 °C/min, the second was 2 °C/min. The dashed line shows the thermal cycle of Experiment 1 until point T1.3 for comparison.

The printed specimens had been thermally treated in a furnace, Hobersal 12-PR/400 (Hobersal, Barcelona, Spain), in a post-process. The study was carried out in two stages. In the first stage, the study covered a wide range of temperatures. Figure 2 shows the temperatures, holding times and heating rates used in the first thermal cycle. At points T1.1, T1.2, T1.3, T1.4, T1.5 and T1.6 of the curve (Figure 2), the specimens were tested as indicated in Section 2.3. Based on the results and conclusions drawn from the first experiment, a second experiment was carried out with the conditions indicated in Figure 3. The points analysed in this curve (T2.1, T2.2 and T2.3) allowed obtaining additional information about the dehydration process and the transition process from hemihydrate to anhydrite to know its effect on mechanical properties. Nine specimens were printed for every testing point on the ramp, making a total of 54 cubes in the first experiment and 27 cubes in the second experiment. After the thermal treatments, the specimens were stored in a desiccator to avoid any contact with water vapour.

### 2.3. Equipment and Tests Performed

The same equipment was used for both experiments and the same properties were tested. The dimensions of all specimens were measured using a Mitutoyo micrometre (Mitutoyo, Kawasaki, Japan) with a 0.01 mm accuracy before and after the thermal treatment. Three points were measured in each direction (X, Y and Z) and the average was calculated. The weight of the cubes was also measured before and after treatment using a balance with a 0.01 g measuring accuracy (Cobos CB Complet, Barcelona, Spain). In the following, the surfaces of three, randomly chosen, treated specimens were tested to evaluate their roughness (Ra) using a Mitutoyo Surftest SJ-500 profilometer (Mitutoyo, Kawasaki, Japan). Each surface (XY, XZ and YZ) of the cubes was measured at five different points and the average for each surface was calculated considering the data from all three cubes. The software used was Formtracepak (Mitutoyo, Kawasaki, Japan). The roughness average (Ra) parameter was calculated according to the ISO 4288:1996 standard [34]. Two cubes were put into glasses for 24 h and the glasses were filled with toluene with a density of 0.864 g/cm^3^ and 90% purity. The cubes were completely covered with toluene the whole time. After 24 h, the cubes were taken out, slightly dried with a piece of paper and weighed again. With the weight before and after the storage in toluene, the porosity was calculated using Archimedes method according to the ASTM C373-88 standard [35]. Six cubes (two for each direction) were used to determine the compression strength using a universal testing machine, Servosis ME-402/5 (Servosis, Madrid, Spain). The tested cubes were placed between two metal blocks that had the same area as the specimens. The test velocity was 0.3 kN/s and the specimens were preloaded with 100 N before the test. The maximum force before the material failure was noted and divided by the specimen’s area to calculate the compression strength [36]. The last cube of every testing point was used to determine the different phases and the water content of the treated specimen. Fourier transform infrared spectroscopy (FTIR) was the method used to clarify the temperature effect on the specimens in this study. The infrared spectroscopy, IR spectrum represents the molecular absorption, creating a molecular fingerprint of the sample; in our case, the spectra were registered in molecular absorption. Considering that calcium-sulphate hemihydrate has different functional groups that absorb infrared light at different wavelengths than those of water, we used FTIR. The equipment was a Jasco FT/IR-4700 FT-IR spectrometer (Easton, MD, USA) equipped with an air-cooled deuterated lanthanum α alanine-doped triglycine sulphate (DLaTGS) detector. The attenuated total reflection (ATR) accessory used was an ATR PRO ONE single-reflection ATR accessory incorporating a monolithic diamond crystal (Laser Components, Bedford, NH, USA). The spectra were collected for a scanning range from 400 to 4000 cm^−1^, with a 4 cm^−1^ resolution and 1 min acquisition time. Table 2 shows the test, equipment and methodology applied in this study.

## 3. Results and Discussion

A preliminary analysis of the specimens’ patterns was carried out to identify each phase, as well as the evolution of the dehydration process with the changes in three variables: temperature, holding time and heating rate. This is important to know the best conditions to achieve optimum mechanical properties.

### 3.1. Infrared Analysis

**Figure 4:**
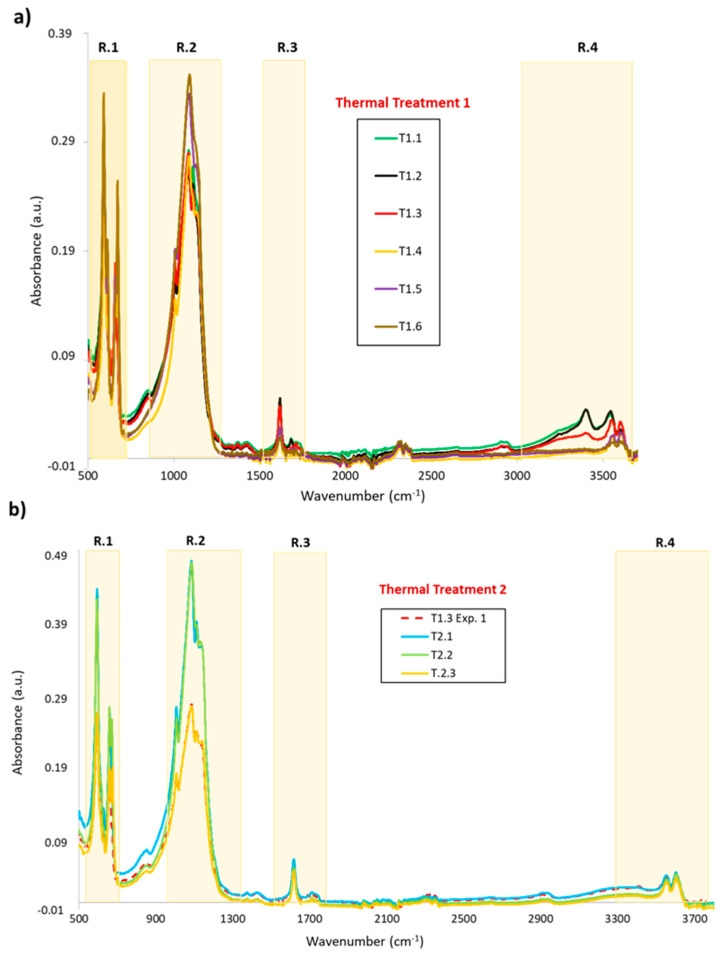
Infrared spectrum showing the four interesting regions for the different testing points for both thermal treatments: (**a**) Experiment 1 and (**b**) Experiment 2.

Figure 4 show the whole IR spectra, ranging from 500 to 3700 cm^−1^, of the specimens for each stage of the thermal treatments. Both IR spectra show vibration modes in four regions, which is interesting for the purposes of this study, to know the dehydration mechanism and the phases present in each step of the heat treatment:

(a) Bending vibration of the SO_4_ tetrahedron in the 550–700 cm^−1^ range
(b) Stretching vibration of the SO_4_ tetrahedron in the 1000–1200 cm^−1^ range
(c) Bending vibration of O–H in the 1500–1700 cm^−1^ range
(d) Stretching vibration of O–H in the 3000–3800 cm^−1^ range

Although the spectra obtained for thermal treatments 1 and 2 have the same regions of interest for water, dehydrate (gypsum), hemihydrate (basanite) and anhydrite, there are some differences in the dehydration process of the CaSO_4_·H_2_O system and the phases present during the heating.

In Table 3 are listed the peak positions of gypsum, bassanite, and anhydrite, associated to the phase of the system and with the vibration modes. These values are in good agreement with those reported in the literature.

#### 3.1.1. Vibrations Bands of the H_2_O Molecule

**Figure 5:**
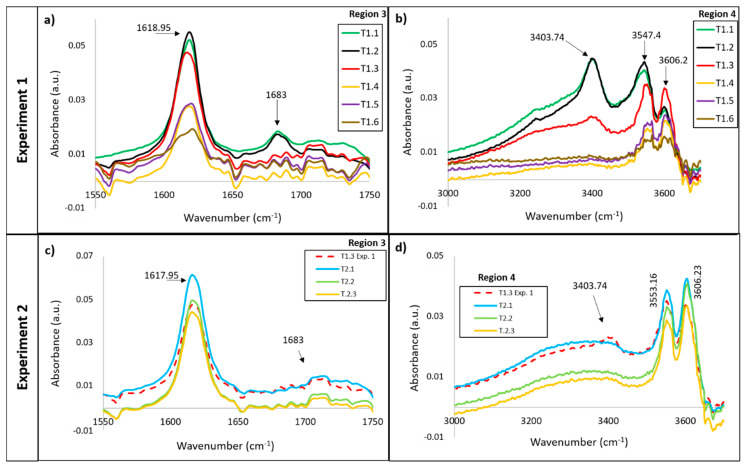
Image of the vibration’s bands of H_2_O molecule for the different testing points for both thermal treatments, showing the amount of water at the different testing points. (**a**) Infrared spectrum between 1550–1750 cm^−1^ for Experiment 1; (**b**) Infrared spectrum between 3300–3700 cm^−1^ for Experiment 1; (**c**) Infrared spectrum between 1550–1750 cm^−1^ for Experiment 2; (**d**) Infrared spectrum between 3300–3700 cm^−1^ for Experiment 2.

The presence of water and the evolution of the dehydration process can be detected by IR because water has five characteristic IR-active modes, two bending: 1618 and 1683 cm^−1^ (Region 3), and three stretching: 3400, 3550 and 3606 cm^−1^ (Region 4) (Figure 5a–d).

For the first thermal treatment, the bending modes of water in Region 3 are attributed to two types of water. One linked by hydrogen bridges (1618 cm^−1^) and the other directly linked to calcium ions (1680 cm^−1^) (Figure 5a). Both vibration bands are present in the gypsum phase. The peak with the lowest frequency and highest intensity is the only one present for the hemihydrate phase. If the evolution of the bending bands is observed with heating, the peak at 1680 cm^−1^, associated with the dehydrate, disappears once the first two heating points have been exceeded. This shows that the water linked to calcium ions disappears between point 2 and point 3. However, the peak associated with basanite is present until the end of the thermal treatment, although it decreases in intensity with the temperature increase. So, the hemihydrate phase amount decreases with the temperature.

Figure 5b shows the infrared spectrum between 3300 and 3700 cm^−1^. The peak at 3400 cm^−1^ is the most important because it is related to the amount of dehydrate, while the peak at 3600 cm^−1^ refers to the amount of hemihydrate. The peak at 3550 cm^−1^ is an overlap of both basanite and gypsum. There are important differences in their evolution with the temperature. The peak at 3400 cm^−1^ is only significant in the first two steps of heating (1.1 and 1.2), which correlates with the high amount of bonded water in the gypsum phase, as does the peak at 1680 cm^−1^. At point 1.3, this peak (1680 cm^−1^) is just slightly present, while at points 1.4 and 1.5, there is no peak. Point 1.3 has a special characteristic of two high peaks at 3550 and 3600 cm^−1^, making it the only point in which a high amount of hemihydrate was detectable.

If these results are compared with those obtained in Treatment 2 (Figure 5c,d), there are some differences. The vibration modes (bending and stretching) associated with the gypsum phase do not appear. In addition, in the stretching bands corresponding to the hemihydrate phase and the mixture (hemihydrate and gypsum), slight changes are observed with increasing temperature.

These observations allow us to draw some conclusions about the dehydration mechanism produced in both heat treatments. The behaviour of water molecules indicates that first there is a loss of water to join to calcium, which indicates that gypsum is dehydrated to hemihydrate in one first step (CaSO_4_·2H_2_O→CaSO_4_·½H_2_O (1)), and then, the hemihydrate loses water to be converted, probably to anhydrite (CaSO_4_·½H_2_O→CaSO_4_ (2)). These results suggest that the best way to obtain the dry specimen is the second treatment. It is better to heat lower rates.

#### 3.1.2. Vibrations Bands of the SO_4_^2−^ Anion

**Figure 6:**
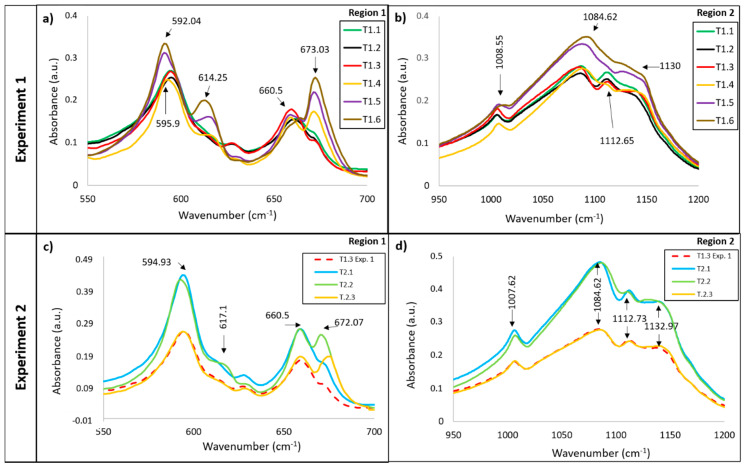
Image of the vibrations bands of SO_4_ anion for the different testing points for both thermal treatments. (**a**) Infrared spectrum between 550–700 cm^−1^ for Experiment 1; (**b**) Infrared spectrum between 950–1200 cm^−1^ for Experiment 1; (**c**) Infrared spectrum between 550–700 cm^−1^ for Experiment 2; (**d**) Infrared spectrum between 950–1200 cm^−1^ for Experiment 2.

As shown in Figure 6a, the only vibration modes that appear during the first three steps of the first heat treatment are at 595 and 660 cm^−1^. These bands correspond to the ν _4_ SO_4_ bending vibration attributed to the hemihydrate phase. After point 1.3, the first band (595 cm^−1^) changes its frequency and intensity. These changes are due to this band splitting into two peaks (592 and 614 cm^−1^), which indicates the formation of anhydrite [39,40]. Also, at this point, a new peak at 673 cm^−1^ appears, which is assigned to anhydrite. The appearance of anhydrite from this point during heating (230 °C, 3 h) corroborates the dehydration mechanism indicated in the previous dehydration process (2). These vibration modes, associated with the non-hydrated phase (the double, 592, 614 and 673 cm^−1^), increase their intensity as the temperature increases. Thus, increasing the amount of anhydrite present in the specimens. However, the 660 cm^−1^ vibration mode assigned to the hemihydrate phase has a contrary behaviour (Figure 6a), which suggests that dehydration is occurring at the expense of anhydrite formation. The changes in the vibration modes produced in this region for the second heat treatment are not so marked (Figure 6c). After the first heating step, there is no significant split of the band at 594 cm^−1^. Although, after point 2, a small shoulder appears, corresponding to the band at 614 cm^−1^ assigned to anhydrite, and a peak of the same phase also appears at 672 cm^−1^, which indicates that a small amount of anhydrite has already formed at 200 °C anhydrite.

The band evolution in Region 2 (Figure 6b,d) is more complicated to describe because these peaks are very weak in the infrared spectra. In this region, peaks appear at 1008, 1084 and 1112 cm^−1^, attributed to basanite, although the last peak could also be attributed to 2–pyrrolidone [33]. Weak changes can be observed with temperature for both treatments. The band at 1112 cm^−1^ disappears after point 1.3 in the first treatment and after point 2.1 for the second treatment. At the same time, a poorly defined and wide absorption band at 1130 cm^−1^ appears, attributed to the presence of the anhydrite phase.

The behaviour of the sulphate anion vibration bands with temperature indicates, as in the water regions, that the dehydration mechanism and the coexistence of phases occur in less time and lower temperatures when using the second treatment. This suggests that the heat rate plays an important role in the dehydration mechanism and anhydrite formation. Both processes seem to produce better for a lower heating rate (2 °C/min) and lower temperature. With the first treatment, it is necessary to heat at 230 °C for 3 h to obtain anhydrite; in the second treatment, this phase appears at 200 °C.

### 3.2. Weight Loss and Porosity

**Figure 7:**
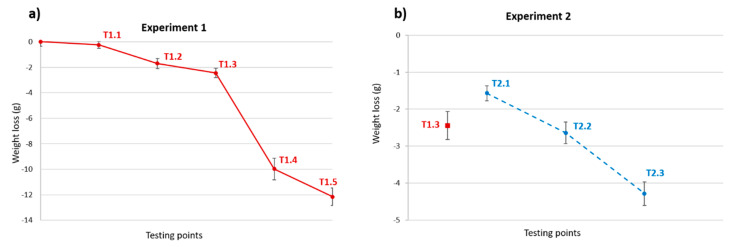
Weight loss in the different testing points for both thermal treatments. (**a**) Experiment 1; (**b**) Experiment 2.

The as-built tested specimens differed in dimension and weight due to the position and the orientation in the printing platform. Several studies have shown that the part orientation and position in the job box affect the densification, and therefore, the weight and dimensional accuracy of the specimens printed [14,15]. The average weight of the specimens was 82.13 ± 2.86 g. Figure 7 shows the weight loss at the different testing points for both experiments. The weight after treatment is less at every point than before the treatment because the water in the material evaporates. At point T1.1, there is only a small weight loss due to the short treatment time (13 min) and low temperature compared to the other points. For points T1.2 and T1.3, the weight loss has a nearly linear tendency, which can be explained by the constant evaporation of the free water in the material. At point T1.4, the weight drops significantly because the applied temperature (230 °C) for a longer time (3 h) caused an additional loss of bonded water (linked to hydrogen bridges) in the material structure. At point T1.5, the rest of this structural water in deeper regions of the specimen evaporates, resulting in an additional weight loss. The curve for Experiment 2 shows a greater weight loss compared to Experiment 1 for similar temperature ranges. This is because a greater dehydration mechanism and anhydrite formation occur due to the lower heating rate, as seen in the infrared analysis.

**Figure 8:**
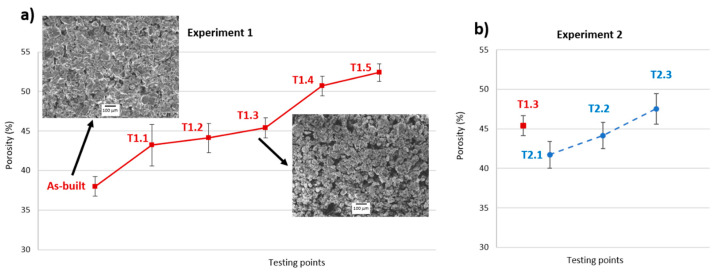
Porosity in the different testing points for both thermal treatments. (**a**) Experiment 1; (**b**) Experiment 2.

The weight loss is related to the increase in porosity experienced by the specimens, as observed in the results of the Archimedes test carried out. From point T1.1 to point T1.3, there is only a small increase in porosity, while at points T1.4 and T1.5, the porosity experienced a more notable increase. Figure 8 shows this trend. Images taken with the Scanning Electron Microscopy (SEM) corresponding to the “as-built” specimen and point T1.3 show the presence of a greater number of gaps between the particles when heat treatments are applied. For Experiment 2, the porosity experienced a linear increase, reaching a value greater than the equivalent for point T1.3 of Experiment 1 at point T2.3. This result indicates that a lower heating rate contributes to the formation of porous structures due to the greater removal of water, which causes the elimination of gypsum and the rapid formation of anhydrite.

### 3.3. Dimensions

**Figure 9:**
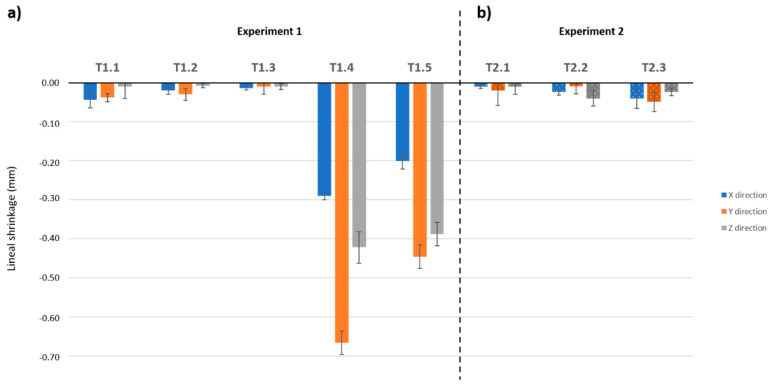
Linear shrinkage in the testing points for both thermal treatments. (**a**) Experiment 1; (**b**) Experiment 2.

The average dimensions were 40.37 ± 0.22 mm in the X-direction, 40.20 ± 0.12 mm in the Y-direction and 40.16 ± 0.10 mm in the Z-direction. These values were used in the following results. Figure 9 shows the variations in the dimensions experienced by the specimens due to heat treatments. For Experiment 1, the dimensions remain almost constant in all directions until point T1.3, with only slight linear shrinkage around 50 μm. This is a result of the evaporation of free water. Points T1.4 and T1.5 experienced a huge shrinkage with clear differences in the different directions. This is a result of the evaporation of structural water, which produces deformation in the material. This deformation is produced as a consequence of physical and chemical changes that occur due to dehydration and the appearance of new phases, generating alterations in the structure. These alterations are related to the appearance of gaps (loss of water molecules), as well as changes in the crystal structure. The adaptation of the sample structure to these changes could translate into a variation of volume, either contraction or expansion. The dimensional variations in Experiment 2 are slightly greater due to the faster rate of dehydration when the heating rate is slower.

### 3.4. Roughness

**Figure 10:**
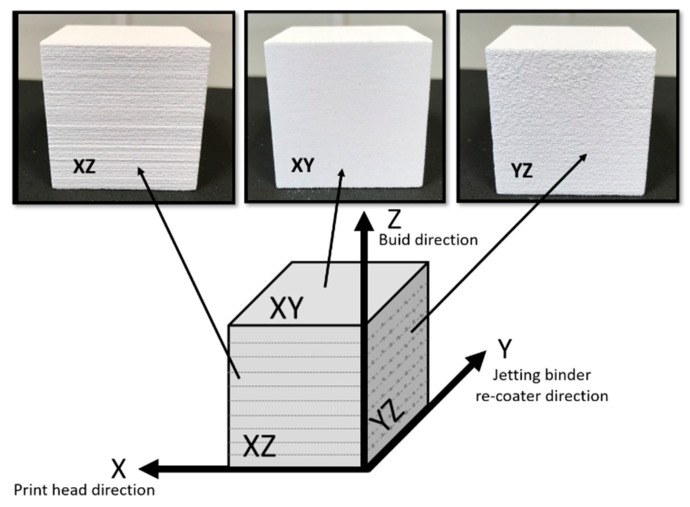
Directions and surface structures of the printed specimens with the Project 660 Pro machine.

The printed specimens showed three different structured surfaces as a result of the printing process. In the printing process, a roller, called a re-coater, moves along the Y-axis of the machine, spreading a 0.1 mm thick layer of powder on the fabrication platform. Next, the print-head moves in the X-axis dropping the binder onto the powder in the appropriate zones according to the part geometry. In this way, the particles are first agglutinated following a line in the X-direction and this process is repeated at different positions on the Y-axis until a layer in the XY plane is completed. The work platform descends a distance equal to the thickness of the layer (0.1 mm) and the process is repeated. As a result of this process, the join between adjacent particles in the same layer (XY surface) is different than the join between adjacent layers. The YZ surface texture is affected by the slight variation of printhead ending points while manufacturing the layers. The XZ surface is from the last line of binder that the printhead made during the process, which results in the clearly visible linear structure. Figure 10 shows the different surface structures.

**Figure 11:**
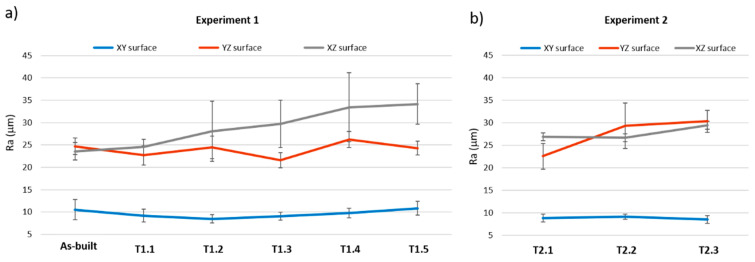
Surface roughness in the testing points for both thermal treatments. (**a**) Experiment 1; (**b**) Experiment 2.

Figure 11 shows the Ra values in the different testing points for both experiments and the different surfaces. The surfaces in the XY plane showed lower roughness with small standard deviations for both experiments. The roughness on this surface does not experience significant changes with heat treatment. In the XY plane, there is no join between the layers and, therefore, the binder and water losses do not significantly affect this surface. Ra values for surfaces in the YZ and XZ planes showed variations and higher standard deviations with heat treatments. The join between layers is involved in these surfaces and, therefore, the blending between the particles is less consolidated, making it more sensitive to water losses and physical changes suffered by the material, causing a greater alteration in these surfaces during heat treatment.

### 3.5. Compression Strength

**Figure 12:**
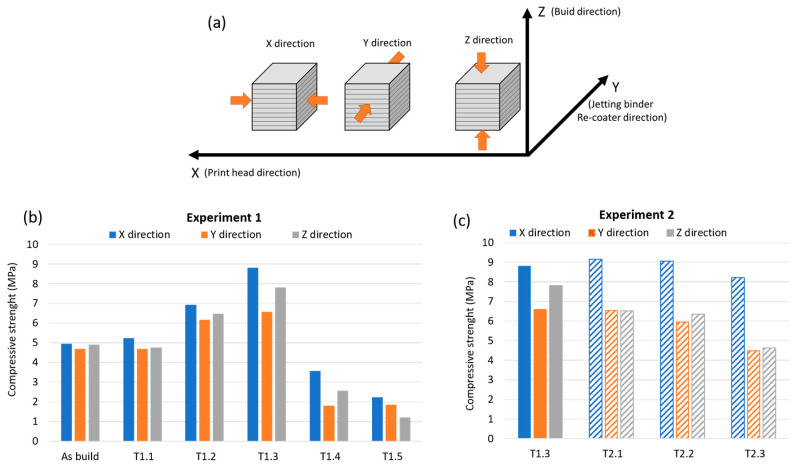
(**a**) Direction of compression force, (**b**) compression strength values for Experiment 1 and (**c**) compression strength values for Experiment 2.

Figure 12 shows the variation of compression strength for different force directions (X, Y and Z) at different testing points for both experiments. The values for the compression strength when the load was applied parallel to the direction of the printhead (X-direction) are the highest. At point T1.3, this difference is more significant. In Experiment 2, this effect is clearly shown for all the tested points. Due to the printhead movement, the join between adjacent particles is different than the join between adjacent layers. This fact explains the anisotropic properties of printed parts. This anisotropic behaviour is in agreement with other studies [6,16].

In Experiment 1, only slight changes of the compression strength occur at point T1.1 for the untreated specimen (as-built). The compression strength then increases significantly for the three directions, reaching maximum values at point T1.3. At points T1.4 and T1.5, the compression strength drops to a value that is even lower than for the untreated specimens. The compressive strength of the material is related to the formation of the different phases that crystallize at different temperatures. The low resistance of the material at points T1.4 and T1.5 is due to the missing structural water, which produces a loss of cohesion among the particles, resulting in the material crumbling. At points T2.1 and T2.2 of Experiment 2, the values are similar. When the temperature rises to point T2.3, a slight decrease in resistance is observed as a consequence of the continuous process of dehydration and formation of anhydrite.

## 4. Conclusions

Under consideration of all results for both experiments, it becomes clear that a post-processing thermal treatment can improve the properties of binder-jetted calcium-sulphate parts. The possibility to achieve higher compression strengths despite the reduction in weight and the higher porosity is significant. Temperatures over 230 °C for a long time are not recommended because the compression strength decreases significantly, and printed parts are affected by shrinkage. It is more important to think about the orientation of parts during the printing process, since it affects the surface quality and compression strength, especially if thermal treatment is applied afterwards.

For the first experiment, point T1.3 showed the best performance. Based on the results and conclusions drawn from the first experiment, a second experiment was carried out. Fourier transform infrared (FTIR) spectroscopy indicated that the dehydration mechanism and the coexistence of phases occur in less time and lower temperatures when using the second treatment. Therefore, we conclude that a lower heating rate (2 °C/min) accelerates the dehydration mechanism and anhydrite formation. The conditions used in the second experiment allowed obtaining parts with properties quite similar to those of point T1.3, especially at point T2.1. The advantage of the second experiment is reducing the highest used temperature from 230 to 180 °C and the reduction of total cycle time from 201 to 126 min, saving energy and time. This research shows the high potential of thermal treatments applied to calcium-sulphate parts manufactured by binder jetting technology.

## Figures and Tables

**Table 1:** Chemical composition impurities in both material: powder and binder.

ICP-OES					
Analite	Mg (285.213)	Na (589.592)	K (766.490)	Al (396.153)	Si (251.611)
CaSO_4_·½H_2_O (mg/kg)	<4	508.26	679.66	<4	253.75
Binder (mg/L)	0.13	73.20	2.24	<0.10	−

**Table 2:** Tests performed to analyse the effect of heat treatments on CaSO_4_·½H_2_O specimens.

Test	Equipment	Methodology
Roughness Measurement	Mitutoyo Surftest SJ-500 Profilometer	ISO 4288:1996 standard
Dimension Measurement	Micrometre Mitutoyo	Three measurements in each direction (X, Y, Z) for each specimen
Weight Measurement	Balance Cobos CB Complet (accuracy of 0.01 g)	Three measurements for each specimen
Porosity	Balance Cobos CB Complet (accuracy of 0.01 g). Toluene.	ASTM C373-88 standard (Archimedes method)
Compression Test	Universal Testing Machine Servosis ME-402/5	EN 13279-2:2014 standard
Composition Analysis	Jasco FT/IR-4700 FT-IR + ATR	IR radiation is passed through the sample

**Table 3:** Observed infrared fundamental modes in cm^−1^ for sulphate anion and water molecule in CaSO_4_·2H_2_O = Dehydrated; CaSO_4_·½H_2_O = Hemihydrate; CaSO_4_ = Anhydrite, from specimen recorded during both thermal treatments.

Wavenumber (cm^−1^) in This Study	Phase of The System CaSO_4_·H_2_O	Corresponding Vibration	References
595.9	CaSO_4_·½H_2_O	ν _4_ SO_4_	[37]
614.25	CaSO_4_	ν _4_ SO_4_	
660.5	CaSO_4_·½H_2_O	ν _4_ SO_4_	
673.03	CaSO_4_	ν _4_ SO_4_	
1008.55	CaSO_4_·½H_2_O	ν _1_ SO_4_	[38]
1084.62	CaSO_4_·½H_2_O	ν _3_ SO_4_	
1112.65 *	CaSO_4_·½H_2_O	ν _3_ SO_4_	
1130	CaSO_4_	ν _3_ SO_4_	
1618.95	CaSO_4_·2H_2_O CaSO_4_·½H_2_O	ν _2_ H_2_O	
1683	CaSO_4_·2H_2_O	ν _2_ H_2_O	
3403	CaSO_4_·2H_2_O	ν _1_H_2_O	
3550	CaSO_4_·2H_2_O CaSO_4_·½H_2_O	ν _3_ H_2_O ν _1_ H_2_O	
3606	CaSO_4_·½H_2_O	ν _3_ H_2_O	

* This peak is attributed to the binder (2–pyrrolidone).
